# Turning waste avocado stones and montmorillonite into magnetite-supported nanocomposites for the depollution of methylene blue: adsorbent reusability and performance optimization

**DOI:** 10.1007/s11356-023-30538-0

**Published:** 2023-11-03

**Authors:** Ahmed S. El-Shafie, Fatima Karamshahi, Marwa El-Azazy

**Affiliations:** Department of Chemistry and Earth Sciences, College of Arts and Sciences, Qatar University, Doha, 2713 Qatar

**Keywords:** Waste avocado stones, Montmorillonite, Magnetite-supported adsorbents, Methylene blue, Central composite design, Reusability

## Abstract

The existence of methylene blue (MB) in wastewater even as traces is raising environmental concerns. In this regard, the performances of four adsorbents, avocado stone biochar (AVS-BC), montmorillonite (MMT), and their magnetite Fe_3_O_4_–derived counterparts, were compared. Results showed the superior performance of Fe_3_O_4_@AVS-BC and Fe_3_O_4_@MMT nanocomposites with removal percentages (%*R*) of 95.59% and 88%. The morphological features of AVS-BC as revealed by SEM analysis showed a highly porous surface compared to a plane and smooth surface in the case of MMT. Surface analysis using FT-IR and Raman spectroscopies corroborated the existence of the Fe–O peaks upon loading with magnetite. The XRD analysis confirmed the formation of cubic magnetite nanoparticles. The adsorption process in the batch mode was optimized using central composite design (CCD). Equilibrium and kinetic isotherms showed that the adsorption of MB onto Fe_3_O_4_@AVS-BC fitted well with the Langmuir isotherm and the pseudo-second-order (PSO) model. The maximum adsorption capacity (*q*_*m*_) was 118.9 mg/g (Fe_3_O_4_@AVS-BC) and 72.39 mg/g (Fe_3_O_4_@MMT). The Fe_3_O_4_@AVS-BC showed a higher selectivity toward MB compared to other organic contaminants. The MB-laden adsorbent was successfully used for the remediation of Cr (III), Ni (II), and Cd (II) with removal efficiencies hitting 100% following thermal activation.

**Supplementary Information:**

The online version contains supplementary material available at 10.1007/s11356-023-30538-0.

## Introduction

The ever-growing industrial development has brought about considerable amounts of pollutants that negatively impacted the ecosystem. Dyes are among these pollutants that are hard to remediate using traditional treatment techniques (Modi et al. 2022, Rafatullah et al. 2010, Yaseen &Scholz 2019). Being considerably used in numerous industries, for instance, cosmetics, pharmaceuticals, textile, food, and beverage, the existence of dyes in wastewater is becoming a serious apprehension. To be able to grasp the magnitude of the problem, it is enough to say that the number of commercially available dyes produced annually exceeds 0.1 million and that the amount of dyes wasted each year represents ~ 5–10% of the produced amount (Benkhaya et al. 2020, Bulgariu et al. 2019, El Messaoudi et al. 2022, Khan et al. 2022, Nipa et al. 2023).

Methylene blue (MB) (Table S1) is a phenothiazine derivative which is freely soluble in water forming a stable solution at room temperature. MB has been involved in a variety of applications, including tannery industries, as a biological stain, for treatment of toxicity following the ingestion of poisonous chemicals, for treatment of malaria, etc. (Khan et al. 2022). With this wide range of applications, the presence of MB in water effluents cannot be ignored (El-Azazy et al. 2021b, Lv et al. 2022, Viscusi et al. 2022). Negative impacts of MB include elevated heartbeat rate, renal failure, and various GIT disorders such as nausea, vomiting, and diarrhea (Khan et al. 2022). Therefore, and with the limited biodegradability, there is a need to create an effective and ecologically acceptable method for eliminating MB from wastewater.

In the course of wastewater treatment, the highly complex, putrescible organic materials are partially eliminated. Unfortunately, this degree of treatment has increasingly shown to be insufficient to produce reusable water. The removal of dyes has been approached through traditional biological, physical, and/or chemical treatments (Bal &Thakur 2022, Kaczorowska et al. 2023, Ruan et al. 2019, Tee et al. 2022). By and large, most of these methods have revealed good performance and high removal capacity for dyestuffs; howbeit, their usage is encumbered by the high technical requisites, elevated cost, difficulty to scale-up, and secondary pollution. On the other hand, the complex structure of MB limits the relevancy of the chemical and biological methods for its degradation. Being of low cost, easy to design and apply, and producing sludge-free effluents, adsorption is among the physical/chemical treatment approaches that are widely used for wastewater remediation (Ambaye et al. 2021, Crini et al. 2019, El-Shafie et al. 2021, Li et al. 2019, Tee et al. 2022). Table 1 shows some of the reported efforts used for the removal of MB from different water matrices using various natural and synthetic adsorbents.

**Table 1:** Reported efforts for the removal of MB from different water matrices

Adsorbent	Water matrix	Initial [MB], (mg/L)	*q*_*m*_/*q*_*e*_ (mg/g)	Removal (%)	Ref
Fe_3_O_4_@AVS-BC	Synthetic wastewater	5–30	118.9	95.59	Current study
Fe_3_O_4_@MMT			72.39	88	
Rhamnolipid-functionalized graphene oxide hybrid	Ultrapure water (pH 6.0)	100, 200	234.40, 331.21	93.76, 66.24	Wu et al. (2014)
	Tap water (pH 7.2)		248.48, 468.55	99.39, 93.71	
	River water (pH 7.4)		248.82, 476.05	99.53, 95.21	
	Dye wastewater (pH 8.3)		249.36, 497.80	99.75, 99.56	
Black olive stones	Textile laundry wastewater	600	458	76.33	Al-Ghouti & Al-Absi (2020)
Green olive stones			525	81.45	
Mango seed kernel powder	Synthetic wastewater	50–250	58.08	NS	Senthil Kumar et al. (2014)
Magnetic manganese oxide halloysite composite	Synthetic wastewater	100	96.47	NS	Zhang et al. (2023a)
Magnetic zeolite	Synthetic wastewater	4–20	9.6	NS	Wang et al. (2022)
MnO_2_@reduced graphene oxide	Synthetic and textile wastewater	30 (synthetic)	156.25	NS	Munonde et al. (2023)
		2.34–3.19 (textile)			
Fe-kepok banana peel activated carbon	Synthetic wastewater	50	NS	94.86	Setiawan et al. (2023)
Modified magnetic chitosan microsphere	Synthetic wastewater	30–80	5.04	80.64	Rahmi et al. (2023)
Hydroxyapatite nanoparticles	Synthetic wastewater	5–90	38.93	88.88	Aaddouz et al. (2023)
Waste tea-activated carbon	Synthetic wastewater	20–100	97.6	89.20	Mariah et al. (2023)

*NS:* not stated

Surveying the literature shows that several materials were reported as efficient adsorbents for wastewater treatment (Abdellaoui et al. 2019, Dutta et al. 2021, El-Azazy et al. 2020, Vu &Wu 2022). Lignocellulosic biomasses are among the commonly explored materials. Thanks to their lignocellulosic structure, biomasses possess a functional group-rich surface that can facilitate scavenging pollutants. Their low cost, availability, and biodegradability are the main pros. Furthermore, recycling of biomasses into valuable products hands round to minimalize waste materials and hence the load on the ecosystem (Asemave et al. 2021, El-Azazy et al. 2021a, Ouyang et al. 2020, Peng et al. 2020, Van Tran et al. 2022).

For the current treatise, waste of avocado stones (AVSs) was selected as a biochar source (AVS-BC). The worldwide annual production of avocado exceeds 6.4 × 10^6^ t. The stone (comprising the seed) constitutes 14–24% of the fruit, and the rest of the fruit is the peel and the pulp (García-Vargas et al. 2020, Kang et al. 2022). By and large, composting services do not accept the AVS which is hard to grind. Therefore, recycling the stones into BC for wastewater remediation is an alternative pathway for alleviating the burden on the ecosystem.

Enhancing the adsorptive capacity of the BC could be done via decoration with metal oxides. Among the metal oxides, magnetite (Fe_3_O_4_)-modified BC nanocomposites are commonly used for wastewater treatment (El-Shafie et al. 2023, Li et al. 2020, Prabakaran et al. 2022, Yi et al. 2020). The existence of magnetite on the surface helps to boost the surface area and hence the prevalence of effective binding sites. Moreover, the improved magnetism imparted by the presence of magnetite facilitates the removal of organic pollutants. In the same context, montmorillonite (MMT), a clay with high surface area and superior cation-exchange capacity, has been decorated with magnetite–Fe_3_O_4_@MMT and used for remediation of MB (Al Kausor et al. 2022, França et al. 2022, Tong et al. 2020).

The current study aims to compare the adsorptive capacity of the naturally derived adsorbent, Fe_3_O_4_@AVS-BC, with the modified clay, Fe_3_O_4_@MMT toward MB. Cost-effectiveness, availability, and adsorption capacity have been considered while evaluating the performance of both adsorbents. In a parallel context, the current study employs a response surface methodology–based approach: the central composite design (CCD) to control the variables affecting the adsorption process. This approach seeks to reduce both the number of experimental runs and the associated consumption of hazardous materials, subsequently minimizing waste generation. In a subsequent step, the capability of the calcinated adsorbent-adsorbate complex to eliminate a different set of pollutants, heavy metals, from wastewater has been explored.

## Materials and methods

### Materials, equipment, and software

MB was obtained from BDH Chemicals Ltd. (UK). Other chemicals such as methyl orange, heavy metals nitrates, NaOH, HCl, NaCl, and montmorillonite K10 (K-catalyst, surface area 250 m^2^/g) (MMT) were all acquired from Sigma-Aldrich (USA). Drug materials used in the selectivity testing (acyclovir, amantadine, raltegravir, econazole nitrate, procaine HCl, and sulfisoxazole) were procured from Biosynth^®^ Carbosynth Ltd. (UK). Deionized water (18.2 MΩ·cm) was acquired from a Millipore-Q system. Avocado stones were gathered from the local eateries in Doha, Qatar. The stones were dried out in an oven (Memmert, GmbH + Co. KG, Germany), powdered using a high-speed multi-function mixer (RRH-1000A, 50-300 mesh, China), and pyrolyzed in a Thermolyne^TM^ furnace (USA) into (AVS-BC).

A stock solution of MB (400 mg/L) was prepared in deionized water and subsequently diluted to concentrations in the range of 5–30 mg/L. To adjust the pH of the water in which the adsorbents were suspended, 0.1 M aqueous solutions of either NaOH or HCl were utilized. The pH values were determined using a Jenway 3305 pH meter (UK). For the measurement of MB at pH values of 2.0, 6.0, and 10.0 ± 0.2, three calibration curves were created. The adsorbent-adsorbate mixture was equilibrated by shaking in an incubator (Stuart, SI500, UK). A UV-visible spectrophotometer (Agilent diode-array, USA) was used to quantify the concentrations of MB before and after adsorption using 10-mm matched quartz cuvettes. Separation of the filtrate was achieved using 0.45-μm Millex membrane filters.

The functional groups on the surface of the adsorbent were identified using FT-IR spectroscopy (PerkinElmer, USA). CHN elemental analysis was done using Thermo Scientific™ FLASH 2000 CHNS/O analyzer (USA). The surface morphology of the adsorbent was investigated using scanning electron microscopy (SEM, FEI, Quanta 200, Thermo Scientific™, USA) and energy dispersive X-ray diffraction (EDX). The thermal stability of the adsorbent was ascertained using thermogravimetric analysis (TGA). Raman spectroscopy was used to investigate the nature of the carbonaceous compound (Thermo Scientific™, USA). The X-ray diffraction (XRD) analysis was conducted using an X-ray diffractometer (X’Pert-Pro MPD, PANalytical Co., the Netherlands) with a Cu Kα X-ray source (λ = 1.540598 Å). Measurements were taken over a 2θ range of 5–90°.

The reusability of the MB-laden Fe_3_O_4_@AVS-BC composite was evaluated versus a mixture of heavy metals. The quantity of heavy metals still present in the filtrate after adsorption onto the calcinated sample was determined by ICP-OES (Optima 7300 DV, PerkinElmer, USA).

### Preparation of avocado stone biochar (AVS-BC)

Stones were removed from the avocado fruit and cleaned up with tap water three times before being washed up three more times with deionized water to remove any dirt or contaminants. To dry the stones, they were placed in the oven for 3 days straight at 70 °C. A high-speed, multi-purpose mixer was then used to ground the stones. The resulting powder was split into two portions. The first portion was designated as “avocado stone-raw” (AVS-R). The second portion was sealed into porcelain crucibles and heated to 600 °C for 60 min. The product was further ground and stored for later use in sealed vials with the designation (AVS-BC).

### Preparation of magnetized adsorbents

Using the co-precipitation method, magnetite (Fe_3_O_4_) nanoparticles were prepared, where 200 mL of 0.1 M Fe^3+^ was combined with 100 mL of 0.1 M Fe^2+^ solution, 200 mL of deionized water were added, and the mixture was stirred at a speed of 700 rpm (Fadli et al. 2019, Petcharoen &Sirivat 2012). A mass of 10.0 g of the AVS-BC or MMT was added to the combination and stirred for 2 h at 70 °C. A few milliliters of NaOH were gradually added to the mixture to adjust the pH to ~ 12. The mixture was washed 10 times with deionized water then with methanol 5 times, and the mixture was filtered under vacuum. The magnetized adsorbent (Fe_3_O_4_@AVS-BC and Fe_3_O_4_@MMT) was dried at 70 °C for 12 h and then sealed in tightly closed vials for subsequent application (Ali et al. 2021, El-Shafie et al. 2023).

### Determination of the point of zero charge (pH_PZC_)

A total of seven volumetric flasks were used in which 50 mL of 0.01 M NaCl was added, followed by a mass of 0.20 g of the adsorbent (AVS-BC, Fe_3_O_4_@AVS-BC, MMT, and Fe_3_O_4_@MMT). The pH of each flask was adjusted to a range of 2.0 to 10.0 ± 0.2 using aqueous solutions of 0.1 M HCl or 0.1 M NaOH. Samples were shaken at 150 rpm for 48 h prior to measuring the final pH. The pH_PZC_ value is the point on the curve where pH_initial_ versus pH_final_ overlaps (Babić et al. 1999).

### Batch adsorption experiments (central composite design (CCD))

The CCD was used in the current study to optimize the adsorption process variables. The preceding design is a 2-level full-factorial design (FFD). The pH (A), adsorbent dose (AD, B), MB concentration ([MB], C), and contact time (CT, D) were the four factors that were looked at (Table 2). The assessed response was the %*R*_MB_ and was calculated using Eq. (1).

1 $$\%R=100\times \frac{C_o-{C}_e}{C_o}$$

**Table 2:** Variables with levels for the CCD, both independent and independent

Variables and their codes	− 1	0	+ 1
pH (pH unit), A	2	6	10
Dose (dose, mg/13 mL), B	20	70	120
Dye concentration ([MB], mg/L), C	5	17.5	30
Contact time (CT, min), D	10	65	120
Dependent variable	Removal percentage, %*R*		

where *C*_*0*_ and *C*_*e*_ are used to indicate the initial and equilibrium concentrations of MB (mg/L), respectively.

The design scenario entailed 30 runs that were performed over 3 blocks. Conducted experiments included 16 cube points, 4 central points (Ct Pt), 8 cube axial points, and 2 Ct Pt in the axial. The CCD was applied twice: once for Fe_3_O_4_@AVS-BC and the second for Fe_3_O_4_@MMT. The scenario of the CCD is exhibited in Table 3. Each run was repeated thrice, and the average %*R* was taken as the measured response. Predicted responses were calculated using the Minitab^®^ software. An assessment of the obtained (experimental) versus predicted values was held, and judgment was based on the percent error (%Er) calculated using Eq. (2).

2 $$\%\mathrm{Er}=100\times \left|\frac{\%{R}_{\mathrm{Pre}}-\%{R}_{\mathrm{Exp}}}{\%{R}_{\mathrm{Pre}}}\ \right|$$

**Table 3:** CCD experimental setup arranged based on the run order. Experimental and predicted %*R*_MB_ are shown

Run order	Blocks	Variables				Fe_3_O_4_@AVS-BC			Fe_3_O_4_@MMT		
		pH	Dose	[MB]	CT	%*R*_Exp_	%*R*_Pre_	%Er	%*R*_Exp_	%*R*_Pre_	%Er
01	3	6	70	30.0	65	88.66	88.42	0.27	77.77	75.23	3.38
02	3	6	20	17.5	65	83.27	83.85	0.69	56.62	52.50	7.85
03	3	6	70	17.5	120	90.86	90.65	0.23	88.11	83.18	5.93
04	3	6	120	17.5	65	92.77	93.11	0.37	86.13	83.48	3.17
05	3	2	70	17.5	65	87.58	87.90	0.36	62.31	59.27	5.13
06	3	10	70	17.5	65	90.33	90.87	0.59	86.99	82.78	5.09
07	3	6	70	17.5	65	90.36	90.64	0.31	79.92	75.64	5.66
08	3	6	70	17.5	10	90.33	91.36	1.13	70.95	68.33	3.83
09	3	6	70	17.5	65	91.07	90.64	0.47	81.86	75.64	8.22
10	3	6	70	5.0	65	81.96	83.35	1.67	67.14	62.66	7.15
11	2	6	70	17.5	65	90.29	90.64	0.39	71.69	75.64	5.22
12	2	10	120	5.0	10	76.98	76.57	0.54	60.63	62.41	2.85
13	2	2	20	5.0	10	74.30	74.34	0.05	13.48	14.31	5.80
14	2	10	20	30.0	10	84.63	84.23	0.47	56.94	59.83	4.83
15	2	10	20	5.0	120	82.46	81.99	0.57	68.38	70.96	3.64
16	2	2	120	30.0	10	93.54	93.80	0.28	75.05	77.77	3.50
17	2	2	120	5.0	120	84.80	85.12	0.38	67.26	69.27	2.90
18	2	2	20	30.0	120	56.70	57.43	1.27	36.28	38.70	6.25
19	2	6	70	17.5	65	91.67	90.64	1.14	72.61	75.64	4.01
20	2	10	120	30.0	120	93.27	93.20	0.08	72.00	75.44	4.56
21	1	2	20	5.0	120	74.43	73.68	1.02	18.72	19.86	5.74
22	1	10	20	5.0	10	84.56	84.21	0.42	49.78	51.49	3.32
23	1	10	20	30.0	120	83.90	84.14	0.29	74.43	74.89	0.61
24	1	6	70	17.5	65	90.54	90.64	0.11	74.67	75.64	1.28
25	1	10	120	5.0	120	71.93	71.55	0.53	83.94	85.82	2.19
26	1	2	120	5.0	10	87.65	86.74	1.05	55.09	56.75	2.93
27	1	2	20	30.0	10	53.72	51.51	4.29	34.12	34.53	1.19
28	1	10	120	30.0	10	94.38	94.19	0.20	58.41	58.53	0.21
29	1	6	70	17.5	65	90.45	90.64	0.21	75.12	75.64	0.69
30	1	2	120	30.0	120	94.34	94.03	0.33	85.77	86.10	0.38

### Equilibrium and kinetics investigation

A 400 mg/L stock solution of MB was made in deionized water. Samples were prepared using suitable dilutions in the same solvent and were in the range of 5–200 mg/L. Using 0.1 M HCl, the pH was tuned to 6.0 ± 0.2. A quantity of 0.100 ± 0.005 g Fe_3_O_4_@AVS-BC was inserted into 13 mL of the prepared samples. Obtained suspensions were kept in a shaking incubator at 150 rpm for 24 h. After that, the solutions were filtered, and the absorbances of the MB samples were determined at 663 nm. The same procedures were followed in the case of the Fe_3_O_4_@MMT.

To examine the adsorption kinetics, 200.0 mL of 100 mg/L MB solution and 0.500 ± 0.005 g of Fe_3_O_4_@AVS-BC were combined and placed on a magnetic stirrer. An aliquot of 10 mL was taken regularly over a period of 120 min. Following each removal, the solution was filtered, and the absorbance for MB was determined at 663 nm. The same procedures were followed in the case of Fe_3_O_4_@MMT.

### Adsorbent-adsorbate composite recyclability

To test the recyclability of the adsorbent-adsorbate mixture left over after the adsorption process, an amount of 1.000 ± 0.001 g of the MB-laden adsorbent was calcinated for 30 min at 500 °C in sealed crucibles in the furnace. A 100 mg/L stock solution of Cd (II), Cr (III), and Ni (II) mixture and further dilutions were made in deionized water. Next, an amount of 0.100 g of the calcined adsorbent-adsorbate mixture was mixed with 20 mL of the 100 mg/L mixture of the heavy metals and then stirred at 150 rpm in the shaker for 30 min. Suspension was then filtered, and the metal concentration was determined using the ICP-OES. The %*R* of the tested metals was determined using Eq. (1).

### Selectivity of the synthesized adsorbent

To test the adsorbent selectivity, the performance of Fe_3_O_4_@AVS-BC toward MB was compared with its performance toward other dyes such as methyl orange and six other organic pollutants possessing different chemical structures: acyclovir, amantadine, raltegravir, econazole nitrate, procaine hydrochloride, and sulfisoxazole (Cantarella et al. 2019, El-Shafie et al. 2023). Chemical structures, stability, and pK_a_ values of the suggested interferents are exhibited in Table S2. Selectivity testing was performed by mixing 13 mL of 50 mg/L from proposed interferents with 0.100 ± 0.005 g of Fe_3_O_4_@AVS-BC. Using a few drops of 0.1 M aqueous solution of HCl, the solutions’ pH was then fixed to 6.0 ± 0.2, and the suspension was left in the shaker at 150 rpm for 30 min. Samples were filtered, and the absorbance was noted at the λ_max_ of each interferent.

## Results and discussion

The study aimed to assess the effectiveness of four different adsorbents, namely, AVS-BC, Fe_3_O_4_@AVS-BC, MMT, and Fe_3_O_4_@MMT, toward the remediation of MB. The obtained results are shown in Table S3, and the removal efficiency (%*R*) was calculated using Eq. (1). The experimental findings indicate that Fe_3_O_4_@AVS-BC and Fe_3_O_4_@MMT exhibited a higher adsorption efficiency toward MB, with %*R* values of 72.28% and 52.85%, respectively, as compared to the AVS-BC and MMT. Accordingly, both adsorbents impregnated with Fe_3_O_4_ nanoparticles were selected in this work for the remediation of MB.

### Characterization of the tested adsorbents

#### SEM, EDX, and CHN analyses

**Fig. 1:**
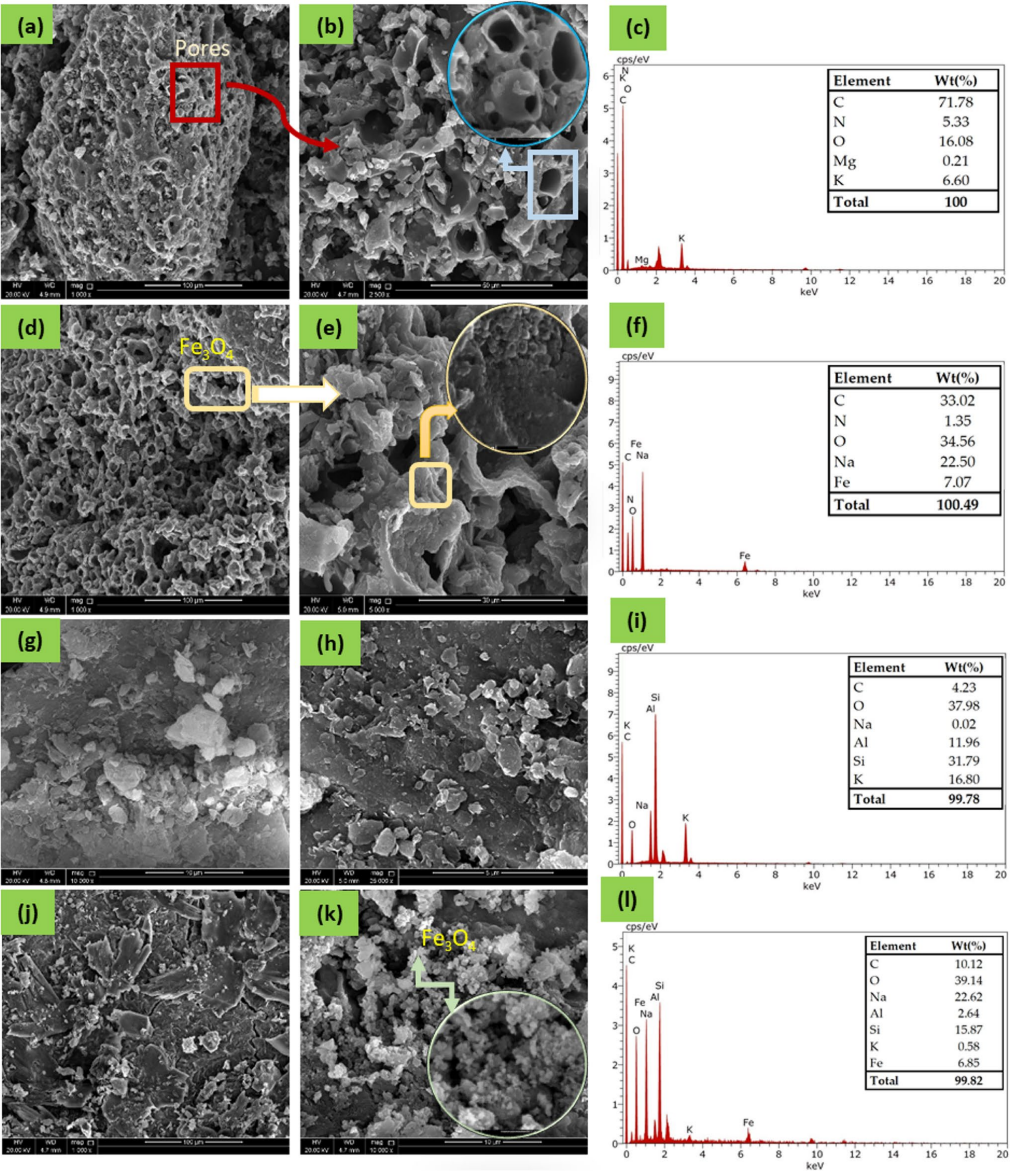
SEM micrographs of (a, b) AVS-BC, (d, e) Fe_3_O_4_@AVS-BC, (g, h) MMT, and (j, k) Fe_3_O_4_@MMT at different magnifications, (c, f, i, l) EDX analyses of AVS-BC, Fe_3_O_4_@AVS-BC, MMT, and Fe_3_O_4_@MMT, respectively

SEM micrographs were obtained for AVS-BC, Fe_3_O_4_@AVS-BC, MMT, and Fe_3_O_4_@MMT, as shown in Fig. 1. For AVS-BC (Fig. 1(a), (b)) the SEM images display a highly porous and irregular surface morphology. The surface of the AVS-BC is highly irregular, with numerous cracks and pores of varying sizes. This highly porous structure of AVS-BC could increase the surface area of the adsorbent and positively affect MB adsorption. On the other hand, the SEM micrographs for the magnetite-impregnated sample (Fe_3_O_4_@AVS-BC) illustrated in Fig. 1(d), (e) show that the surface morphology is identical to that of the AVS-BC samples, with a highly porous and irregular surface structure. It also shows the existence of magnetic nanoparticles on the biochar surface and inside the pores of the AVS-BC structure. The magnetite nanoparticles loaded onto AVS-BC are uniformly distributed on the surface, forming a layer of magnetic nanoparticles. The SEM micrographs, therefore, confirm the successful impregnation of the AVS-BC with magnetite nanoparticles.

The morphological structure of MMT before and after loading with magnetic nanoparticles is exhibited in Fig. 1(g), (h). As shown by the SEM micrographs, the surface of the MMT is plane, smooth, and not an amorphous structure, which could decrease the surface area, an issue which could affect the adsorption efficiency of MMT toward MB. Besides, the magnetite-loaded MMT (Fig. 1(j), (k)) typically shows the existence of irregularly shaped Fe_3_O_4_ nanoparticles dispersed on the surface or intercalated within the interlayer spaces of the MMT clay. The presence of Fe_3_O_4_ nanoparticles could modify the surface area, surface charge, and adsorption properties of the MMT clay.

EDX analysis further validated the SEM observations. The EDX spectra in Fig. 1(c), (f) correspond to AVS-BC and Fe_3_O_4_@AVS-BC, respectively, and revealed a decrease in the %carbon content from 71.78% in AVS-BC to 33.02% in Fe_3_O_4_@AVS-BC. This decrease was attributed to the presence of Fe on the surface of the biochar. Additionally, the %oxygen content increased from 26.08% in AVS-BC to 34.56% in Fe_3_O_4_@AVS-BC due to the constitution of Fe_3_O_4_ nanoparticles on the biochar surface. The presence of magnetite was also confirmed by detecting 22.50% Fe in the Fe_3_O_4_@AVS-BC spectrum. Similarly, the EDX spectra for MMT and Fe_3_O_4_@MMT (Fig. 1(i), (l)) revealed a decrease in the %silicon content from 31.79% in MMT to 15.87% in Fe_3_O_4_@MMT, caused by the formation of Fe_3_O_4_ nanoparticles. Furthermore, the presence of magnetite in Fe_3_O_4_@MMT was confirmed by detecting 6.85% Fe in the spectrum. The EDX analysis results provided further evidence for the successful loading of the Fe_3_O_4_ nanoparticles onto the surface of AVS-BC and MMT, which resulted in the modification of their elemental composition.

The results of the CHN analysis are presented in Table S4. The data indicates that the %C in the AVS-BC decreased from 71.12 to 36.17% upon loading with Fe_3_O_4_ nanoparticles to form Fe_3_O_4_@AVS-BC. Conversely, the %C in the MMT sample increased from 0.98 to 1.3% in Fe_3_O_4_@MMT. The low carbon content in MMT can be attributed to its main constituent, silicon. The %H in both adsorbents increased after loading with magnetite, from 1.65% and 1.06% (in AVS-BC and MMT, respectively) to 3.63% and 1.60% in Fe_3_O_4_@AVS-BC and Fe_3_O_4_@MMT, respectively. In contrast, the %N in AVS-BC decreased from 0.15 to 0.12% in Fe_3_O_4_@AVS-BC, while in MMT, the %N increased from 0.05 to 0.10% in Fe_3_O_4_@MMT. The CHN analysis results were consistent with the EDX findings, confirming the accuracy of the data.

#### TGA, Raman, and XRD analyses

**Fig. 2:**
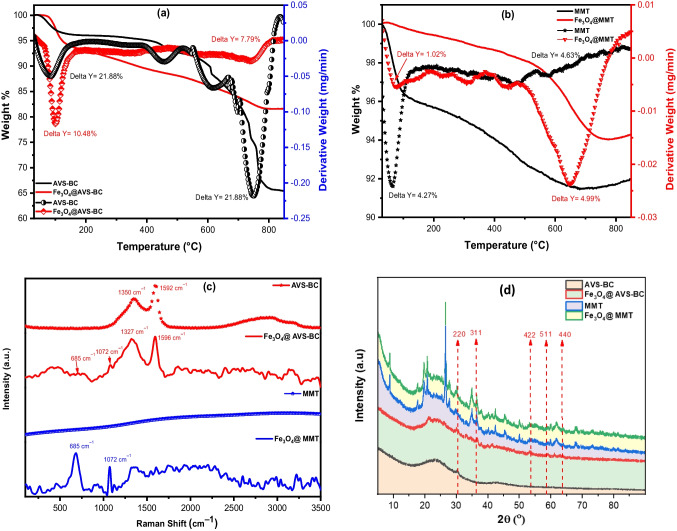
**a** TGA/*d*TA of AVS-BC and Fe_3_O_4_@AVS-BC, **b** TGA/*d*TA of MMT and Fe_3_O_4_@MMT, **c** Raman spectra of the as-prepared samples, and **d** powder XRD pattern of AVS-BC, MMT, Fe_3_O_4_@AVS-BC, and Fe_3_O_4_@MMT

Figure 2 a displays the TGA/*d*TA analysis results for two adsorbents: AVS-BC and Fe_3_O_4_@AVS-BC. The outcomes indicate two weight losses between 40 and 200 °C, where AVS-BC and Fe_3_O_4_@AVS-BC exhibit weight losses of 21.88% and 10.48%, respectively. These losses can be attributed to the evaporation of surface-free water. Additionally, another weight loss was observed in the range of 400–850 °C, where AVS-BC and Fe_3_O_4_@AVS-BC showed weight losses of 21.88% and 7.79%, respectively. This could be due to the carbonization of the polymeric constituents in the carbonaceous material and the loss of organic matter. The total weight loss was 65.24% for AVS-BC and 81.65% for Fe_3_O_4_@AVS-BC, indicating that the existence of magnetite nanoparticles on the surface of AVS-BC enhances the nanosorbent thermal stability. Similarly, the obtained data for the TGA/*d*TA analysis results for MMT and Fe_3_O_4_@MMT are shown in Fig. 2b. The weight loss for MMT and Fe_3_O_4_@MMT between 40 and 200 °C was 4.27% and 1.02%, respectively, which could be attributed to the loss of free water molecules. The second weight loss was found in the range of 450–800 °C, with MMT and Fe_3_O_4_@MMT exhibiting weight losses of 4.63% and 4.99%, respectively, implying that the total weight loss for both samples was around 92.12% and 94.46%, indicating that both samples are thermally stable.

Raman analysis was employed to study the structure of as-prepared Fe_3_O_4_ and both AVS-BC and MMT nanocomposites, as illustrated in Fig. 2c. The spectra exhibited representative Raman modes, the characteristic peaks of the magnetite nanoparticles present in both Fe_3_O_4_@AVS-BC and Fe_3_O_4_@MMT, including the peak at 1072 cm^−1^, which corresponds to the stretching vibration of Fe–O bonds, and the one at 685 cm^−1^ related to the bending vibration of Fe–O bonds, further signifying the presence of magnetite. The obtained data agrees with the reported data for the as-prepared Fe_3_O_4_ nanoparticles (Abd elfadeel et al. 2023, Xie et al. 2020). Alternatively, the Raman spectra for AVS-BC showed the presence of two strong bands at 1592 cm^−1^, which corresponds to a D-band (related to the presence of *sp*^*3*^ C–C atoms), and the second band (G-band) at 1350 cm^−1^, which is called a graphitic band, related to the E_2g_ phonon of *sp*^*2*^ carbon (C–C) atoms, which are characteristic peaks of carbonaceous materials (Chen et al. 2023, Wang et al. 2015, Xu et al. 2015, Zhang et al. 2023b). Additionally, the D-band and G-band were shifted from 1592 cm^−1^ and 1350 cm^−1^ in the AVS-BC to 1596 cm^−1^ and 1327 cm^−1^ in the Fe_3_O_4_@AVS-BC, implying the formation of a bond with the magnetic nanoparticles, which resulted in changing the structure of the as-prepared biochar.

XRD analysis is crucial in determining the crystalline phase of powdered materials. The XRD analysis was performed to verify the crystalline phase of AVS-BC and MMT before and after loading with Fe_3_O_4_ nanoparticles. Figure 2d displays the XRD diffractogram pattern for the prepared samples, including AVS-BC, Fe_3_O_4_@AVS-BC, MMT, and Fe_3_O_4_@MMT. The XRD pattern for the AVS-BC sample displays a broad peak between 2θ 18° and 29°, indicating its amorphous state. This peak was also observed in Fe_3_O_4_@AVS-BC, substantiating the existence of a carbon layer with Fe_3_O_4_ nanoparticles (Elamin et al. 2023, Pravakar et al. 2021). The XRD pattern for Fe_3_O_4_@AVS-BC and Fe_3_O_4_@MMT exhibits three intense peaks at 2θ 30.14°, 36.40°, and 58.15°, which could be attributed to cubic Fe_3_O_4_ (ICDD: 98-015-8743). These findings are consistent with previous reports and confirm the presence of cubic Fe_3_O_4_ nanoparticles on the surface of Fe_3_O_4_@AVS-BC and Fe_3_O_4_@MMT (Mahadevan et al. 2007, Menchaca-Nal et al. 2023, Shirazi et al. 2023).

#### FT-IR analysis and the point of zero charge of the as-prepared adsorbents

**Fig. 3:**
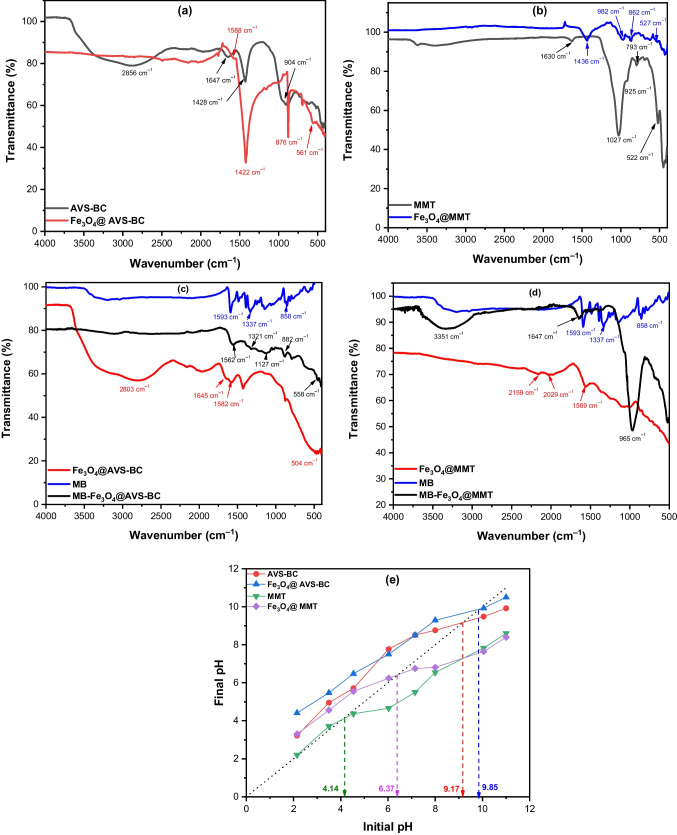
FT-IR spectrum of **a** AVS-BC and Fe_3_O_4_@AVS-BC and **b** MMT, Fe_3_O_4_@MMT, and FT-IR spectrum after MB adsorption onto **c** Fe_3_O_4_@AVS-BC, **d** Fe_3_O_4_@MMT, and **e** pH_PZC_ for the as-prepared samples

The FT-IR spectra of the as-prepared samples AVS and MMT before and after decoration with magnetic nanoparticles are shown in Fig. 3a, b. The IR spectrum of AVS-BC shows the presence of significant absorption bands for biochar functional groups, including a peak at 2856 cm^−1^, fitting to the stretching vibration of C–H bonds in the aliphatic group, such as CH_2_ and CH_3_. Additionally, the band at 1647 cm^−1^ may be related to the C=C stretching vibration in the biochar, and the peak at 1428 cm^−1^ conforms to the presence of bending vibration of –CH_2_ and –CH_3_ groups. On the other hand, the IR spectrum of the Fe_3_O_4_@AVS-BC nanocomposite shows the presence of significant peaks for the magnetic nanoparticles, such as the absorption band at 561 cm^−1^, corresponding to the bending vibration of Fe–O bonds, indicating the presence of magnetite on the surface (El-Azazy et al. 2022, Lan et al. 2022, Liu et al. 2020). Also, the sharp peak at 876 cm^−1^ can be assigned to the bending vibration of Fe–O bonds in the magnetite nanoparticles. Furthermore, it shows the same peaks as AVS-BC, but with a trivial shift, such as the peak at 1428 cm^−1^, which shifted to 1422 cm^−1^ in the nanocomposite, further confirming the presence of magnetite on the surface.

The FT-IR spectrum in Fig. 3b illustrates the significant peaks for MMT and Fe_3_O_4_@MMT samples. The peak at 1630 cm^−1^ corresponds to O–H stretching from water molecules in the interlayer spaces of the MMT, while the broadband at 1027 cm^−1^ indicates the Si–O bending. The peak at 925 cm^−1^ is related to the stretching vibration of Si–O–Si bonds in the tetrahedral sheet of the MMT clay, and the band at 793 cm^−1^ corresponds to the bending vibration of Si–O–Si bonds in the tetrahedral sheet (Jang &Yeo 2015, Jang &Lee 2018). In contrast, the IR spectrum of Fe_3_O_4_@MMT exhibits slight shifts in the functional groups of the MMT clay, indicating the formation of a bond with magnetic nanoparticles. For example, the peak at 1027 cm^−1^ is shifted to 982 cm^−1^. Moreover, the band at 1436 cm^−1^, which corresponds to the bending vibration of –OH groups in the montmorillonite clay structure, appears in the MMT at 1630 cm^−1^. The presence of magnetite nanoparticles can modify the surface charge and adsorption properties of the clay, leading to changes in the intensity and position of this absorption band. Additionally, the peak at 527 cm^−1^ may be attributed to the stretching vibration of Si–O–Si or Fe–O bonds of the magnetic nanoparticles, confirming the presence of magnetite on the surface of the particles.

The FT-IR analysis both before and after the adsorption of MB onto Fe_3_O_4_@AVS-BC (Fig. 3c) indicates a slight shift in the locations of some functional groups due to bonding with the MB dye. Specifically, the peak at 1582 cm^−1^ shifted to 1562 cm^−1^ after adsorption, suggesting the possibility of π-π interactions (Yang &Cannon 2022). Additionally, the peak at 2803 cm^−1^ in Fe_3_O_4_@AVS-BC has disappeared after adsorption, suggesting the occurrence of hydrogen bonding. In addition, the FT-IR spectrum for Fe_3_O_4_@MMT after adsorption of MB (Fig. 3d) shows a shift in the absorption band of MB at 1593 to 1647 cm^−1^. The original band could be attributed to the deformation vibration of the aromatic ring, and the shift could be ascribed to the π-π interactions between MB and the Fe_3_O_4_@MMT adsorbent.

In Fig. 3e, the pH_PZC_ was determined to estimate the surface charge of both MMT and AVS, before and after impregnation with Fe_3_O_4_ nanoparticles. The data obtained revealed that the pH_PZC_ of AVS-BC and Fe_3_O_4_@AVS-BC was 9.17 ± 0.20 and 9.85 ± 0.20, respectively. These results indicate that the surface of Fe_3_O_4_@AVS-BC is negatively charged at pH values higher than 9.85, while at pH values lower than 9.85, it is positively charged. This charge behavior could influence the removal efficiency of MB dye. Regarding MMT and Fe_3_O_4_@MMT, the pH_PZC_ was determined to be 4.14 ± 0.20 and 6.37 ± 0.20, respectively. This data suggests that the surface charge of AVS biochar is mainly positive, while for MMT biochar, it is negative at pH values higher than 4.14 and 6.37 for MMT and Fe_3_O_4_@MMT, respectively.

### Central composite design (CCD) analysis

Like other design-based experiments, utilization of CCD serves to lessen the amount of used chemicals (where a fewer number of runs is conducted), and consequently, the waste to be generated decreases. In addition, the utilization of the design allows the estimation of variable-variable relationships and their impact on the assessed response in almost no time; therefore, the obtained data could be treated with a high degree of certainty (Basheer et al. 2021, Hassan et al. 2020, Heydari et al. 2023). As mentioned, the current design entailed 30 experimental runs as shown in Table 3. As will be detailed in the next subsections, obtained theoretical models were evaluated using the Pareto chart and the analysis of variance (ANOVA).

### Pareto chart

**Fig. 4:**
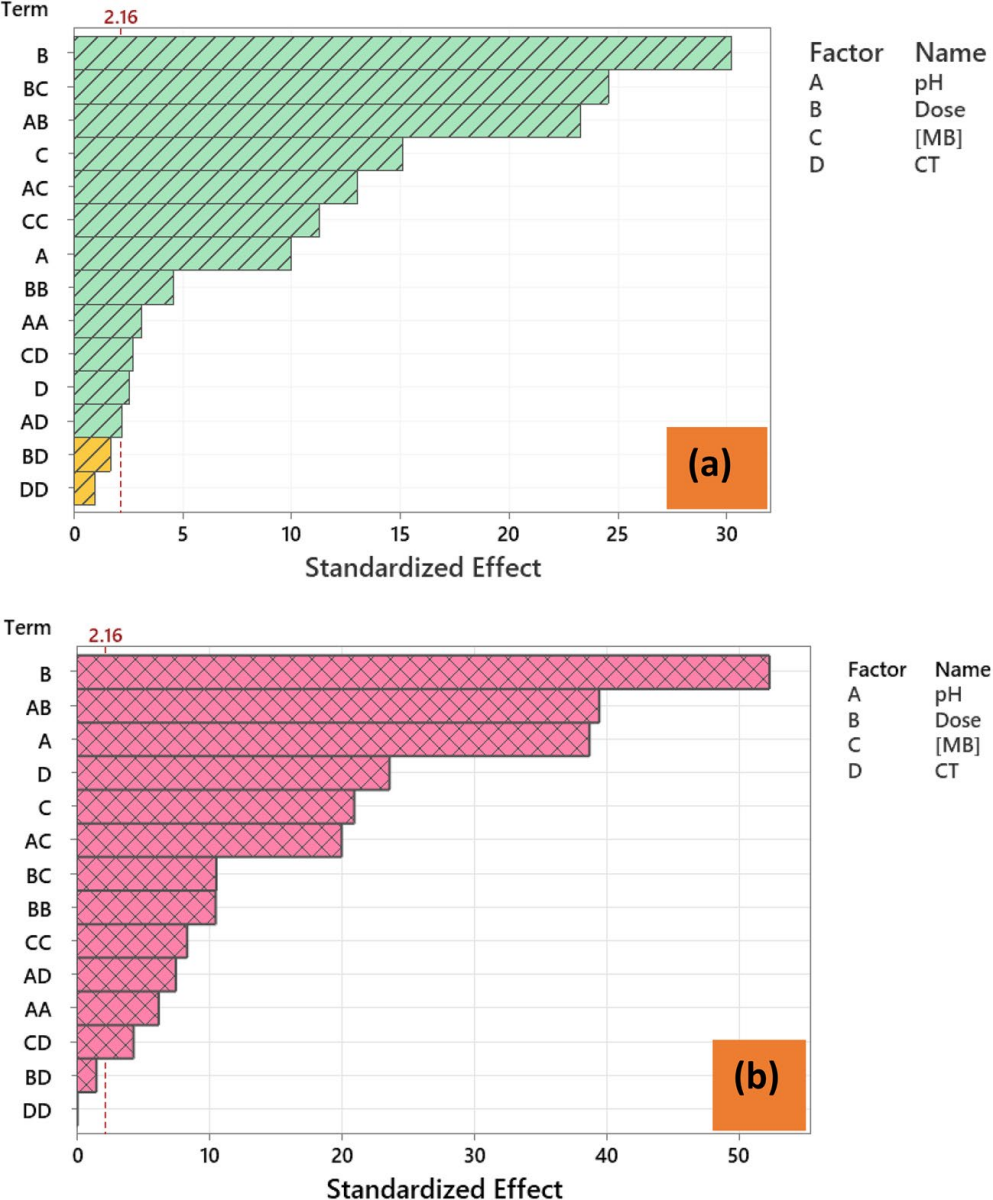
Pareto chart of standardized effect **a** Fe_3_O_4_@AVS-BC and **b** Fe_3_O_4_@MMT

The Pareto chart is a useful tool for determining the importance of the tested factors. The Pareto charts of standardized effects are depicted in Fig. 4 for both Fe_3_O_4_@AVS-BC (a) and Fe_3_O_4_@MMT (b). For both adsorbents, the dose (B) was the most statistically significant main effect when the response is %*R*. Variable-variable interactions of dose × [MB] (BC) and pH × dose (AB) were the second most influential variable. Noticeably, the order of the statistically significant main effect differed in both adsorbents, an issue which could be used later to comprehend the adsorption mechanism on both adsorbents.

### Regression models and ANOVA

Equations in the factorial regression model clearly and thoroughly depict the relationship between dependent and independent variables. This made it simple to determine the total effect of any variable on the observed response using these equations. Equations (3) and (4) were used to describe such a relationship using the coded variables. It is crucial to mention that response transformation was performed using a transformation factor λ = 4 (g = 84.0361 as the geometric mean of %*R*) in the case of Fe_3_O_4_@AVS-BC, Eq. (3), and Box-Cox response transformation with λ = 0.5 in the case of Fe_3_O_4_@MMT, Eq. (4) (Box &Cox 1964).

3 $$\left(\%{R}_{\mathrm{F}e3O4@\mathrm{AVS}-{\mathrm{B}\mathrm{C}}}\right)^{\lambda -1}/\left(\lambda \times {g}^{\left(\lambda -1\right)}\right)=1.99+2.375\ \mathrm{A}+0.2402\mathrm{B}+0.555\mathrm{C}-0.0217\mathrm{D}-0.0933{\mathrm{A}}^2-0.000874{\mathrm{B}}^2-0.03448{\mathrm{C}}^2+0.000155{\mathrm{D}}^2-0.022109\mathrm{AB}+0.04956\mathrm{AC}-0.001942\mathrm{AD}+0.007463\mathrm{BC}-0.000117\mathrm{BD}+0.000750\mathrm{CD}$$

4 $$\surd \%{R}_{\mathrm{Fe}3\mathrm{O}4@\mathrm{MMT}}=0.205+0.7557\mathrm{A}+0.07493\mathrm{B}+0.1969\mathrm{C}+0.00537\mathrm{D}-0.01866{\mathrm{A}}^2-0.000202{\mathrm{B}}^2-0.002575{\mathrm{C}}^2-0.000001{\mathrm{D}}^2-0.003784\mathrm{AB}-0.007674\mathrm{AC}+0.000653\mathrm{AD}-0.000323\mathrm{B}\mathrm{C}+0.000011\mathrm{BD}-0.000120\mathrm{CD}$$

To assess the obtained model, figures such as the coefficient of determination (*R*^2^), the adjusted-*R*^2^ (*R*^*2*^-adj), and the predicted-*R*^2^ (*R*^2^-pred) were perceived and operated to determine the model linearity as well as its predictive potential. The derived models are linear since the *R*^2^ and *R*^2^-adj values are sufficiently high. The *R*^2^-pred values are used to assess a model’s propensity to predict the outcome of a new observation; a high value of (*R*^2^-pred) denotes a suitable level of propensity for the derived regression models. The concordance between experimental and anticipated values is shown by the tiny values of the percent relative error (%Er) (Table 3). ANOVA testing was performed following the response optimization, and the obtained results show an agreement with the findings of the Pareto chart.

### Optimization phase

The 2D contour and the 3D surface plots are displayed in Figure S1. In the contour plots (Figure S1a and b), the response lines are indicated as a function of the levels of two variables, and the dark regions represent zones with maximum response. Taking the upper left panel as an example (Fe_3_O_4_@AVS-BC is the adsorbent), a %*R* ˃ 90% could be achieved using an AD between ~ 90 and 120 mg/13 mL and pH between 3 and 9. In the response surface plots (Figure S1c and d), the response is displayed on the third dimension. The elevated ridge represents a region where the %*R* is maximum. Desirability function, on the other hand, was used to evaluate the effect of tested variables on the measured response based on the obtained value of the individual Derringer desirability function (*d*) and how close to 1.000 (Derringer &Suich 1980). Considering Fe_3_O_4_@AVS-BC as the adsorbent and %*R* as the responses being assessed, a *d-*value of 1.000 was obtained when variables were set at the following levels: a pH of ~ 5, dose of 120 mg/13 mL, [MB] of ~ 25 mg/L, and CT of 10 min. Such a factorial mixture has achieved a %*R* of 95.59%. For Fe_3_O_4_@MMT, a pH of ~ 5, dose of ~ 120 mg/13 mL, [MB] of ~ 28 mg/L, and CT of 112 min could be used to achieve %*R* of 88.10% with a *d-*value of 1.000.

### Equilibrium and kinetics studies

#### Equilibrium investigations

This study investigates the adsorption of MB and the types of adsorbent-adsorbate interactions employing the adsorption isotherms. In this regard, four models were used to analyze the adsorption of MB onto Fe_3_O_4_@AVS-BC and Fe_3_O_4_@MMT: Langmuir, Freundlich, Temkin, and Dubinin-Radushkevich (D-R) (Dubinin M 1947, Freundlich 1907, Langmuir 1918, López-Luna et al. 2019, Sparks 2003, Temkin M 1940, Tonk &Rápó 2022). Model assumptions are summarized in the supplementary file. The equations depicting each model are presented in Table S6.

**Fig. 5:**
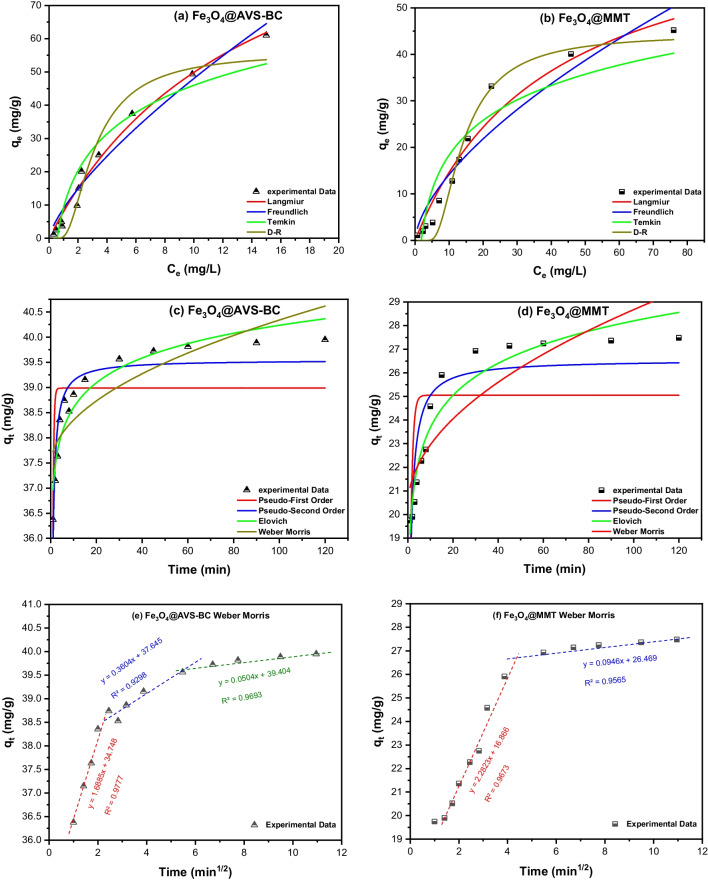
Equilibrium models including Langmuir, Freundlich, Temkin, D-R and kinetic models including PFO, PSO, Elovich, and Weber-Morris for **a** Fe_3_O_4_@AVS-BC and **b** Fe_3_O_4_@MMT. Besides the kinetic models for the adsorption of MB onto **c** Fe_3_O_4_@AVS-BC and **d** Fe_3_O_4_@MMT. Besides the multilinear Weber-Morris model for Fe_3_O_4_@AVS-BC (**e**) and Fe_3_O_4_@MMT (**f**)

Figure 5a, b shows the Langmuir isotherm for the removal of MB using Fe_3_O_4_@AVS-BC and Fe_3_O_4_@MMT, respectively. For both adsorbents, the *R*_*L*_ value was ˂ 1, revealing that the adsorption of MB was favorable. The maximum adsorption capacity (*q*_*m*_) of MB was calculated to be 118.9 mg/g and 72.39 mg/g for Fe_3_O_4_@AVS-BC and Fe_3_O_4_@MMT, respectively, which further validates the results of the CCD analysis where Fe_3_O_4_@AVS-BC has shown better removal efficiency compared to Fe_3_O_4_@MMT. The obtained *R*^2^ values (0.9838 for Fe_3_O_4_@AVS-BC and 0.9599 for Fe_3_O_4_@MMT) suggest that the adsorption of MB onto both adsorbents conformed well to the Langmuir isotherm model. This was further confirmed by the lowest value of the non-linear regression chi-square (*χ*^2^) value (Table 4), calculated using the formula in Table S5.

**Table 4:** Equilibrium and kinetic models’ parameters for the adsorption of MB onto both Fe_3_O_4_@AVS-BC and Fe_3_O_4_@MMT

Model	Parameters	Value	
		Fe_3_O_4_@AVS-BC	Fe_3_O_4_@MMT
Langmuir	*q*_*m*_ (mg/g)	118.9	72.39
	*K*_*L*_ (L. mole^−1^)	0.072	0.025
	χ^2^	7.46	11.35
	*R*^2^	0.9838	0.9599
Freundlich	1/*n*	0.73	0.62
	*K*_*F*_ (mole/g) (L/mole)^1/*n*^	8.99	3.36
	χ^2^	15.41	24.05
	*R*^2^	0.9666	0.9149
Temkin	*b*_*T*_(J/mole)	155.4	229.2
	*A*_*T*_ (L/mole)	1.791	0.545
	*χ*^2^	43.35	42.81
	*R*^2^	0.9060	0.8487
D-R	*β*	3.13 × 10^−9^	5.19 × 10^−8^
	*E* (kJ/mole)	12.64	3.10
	*q*_*m*_ (mg/g)	55.88	44.35
	χ^2^	21.41	17.96
	*R*^2^	0.9536	0.9218
Pseudo-first order (PFO)	*K*_1_ (min^−1^)	2.607	1.059
	*q*_*e*_ (mg/g)	38.98	25.05
	*χ*^2^	0.77	6.99
	*R*^2^	0.4434	0.3301
Pseudo-second order (PSO)	*K*_2_ (g.mg^−1^.min^−1^)	0.239	0.062
	*q*_*e*_ (mg/g)	39.55	26.56
	*χ*^2^	0.16	2.55
	*R*^2^	0.9122	0.9311
Elovich model	*α*	2.87 × 10^22^	3.44 × 10^4^
	*β*	1.408	0.509
	*χ*^2^	0.19	3.72
	*R*^2^	0.8844	0.7552
Weber-Morris (WM) model	*K*_I_	0.291	0.837
	*K*_I1_	1.688	2.282
	*K*_I2_	0.360	0.095
	*K*_I3_	0.050	
	*C*	37.43	20.31
	*χ*^2^	0.41	2.91
	*R*^2^	0.7048	0.7801

All non-linear equilibrium and kinetic equations are listed in Table S6

*χ*^2^ non-linear regression Chi-square

The obtained data of the Freundlich model (Table 4) reveals that Fe_3_O_4_@AVS-BC exhibits a 1/*n* value of 0.73 and an *n* value of 1.37, while for Fe_3_O_4_@MMT, the 1/*n* value is 0.62, and the *n* value equals 1.61. Consequently, Fe_3_O_4_@AVS-BC depicts a higher affinity for MB adsorption compared to Fe_3_O_4_@MMT, indicating its superior adsorption potential.

By analyzing the data obtained from the Temkin model (Fig. 5a, b and Table 4), it was found that Fe_3_O_4_@AVS-BC has an adsorption energy of 155.4 J/mol, while Fe_3_O_4_@MMT has an adsorption energy of 229.2 J/mol. These results suggest that MB molecules can be effectively adsorbed onto the surfaces of both Fe_3_O_4_@AVS-BC and Fe_3_O_4_@MMT nanosorbents. Furthermore, these findings align with the outcomes obtained from the Langmuir and Freundlich models, indicating the reliability of the experimental data.

The obtained results from the D-R model (Table 4) showed that the adsorption energy of MB onto Fe_3_O_4_@AVS-BC is 12.64 kJ/mol, while for Fe_3_O_4_@MMT, it is 3.10 kJ/mol. These findings suggest that the adsorption of MB onto Fe_3_O_4_@AVS-BC could have occurred via chemical ion exchange; thus, the adsorption energy is between 8 and 16 kJ/mol (Chabani et al. 2006, Hu &Zhang 2019), meaning that the adsorption process depends mainly on the presence of the functional groups and the pH of the MB solution. On the other hand, the adsorption of MB onto Fe_3_O_4_@MMT is recognized as physisorption, with adsorption energy lower than 8 kJ/mol, which could have resulted from intermolecular forces such as van der Waals forces.

#### Kinetic investigations

In order to explore the adsorption of MB onto Fe_3_O_4_@AVS-BC and Fe_3_O_4_@MMT, four kinetic models were utilized: pseudo-first-order (PFO), pseudo-second-order (PSO), Elovich, and Weber-Morris (WM) (Amin et al. 2022, Charaabi et al. 2021, Ho &McKay 1999, Lagergren S 1898, Narasimharao et al. 2022, Weber &Morris 1963, Wu et al. 2022) (Fig. 5c, d). The non-linear equations depicting these models are presented in Table S6. The estimated parameters for each model are presented in Table 4. The outcomes suggest that the PSO model is a suitable fit for describing the adsorption of MB onto both Fe_3_O_4_@AVS-BC and Fe_3_O_4_@MMT with *R*^2^ values of 0.9122 and 0.9311, correspondingly and *χ*^2^ values of 0.16 and 2.55, respectively. These results imply that the rate of the adsorption process of MB onto the two adsorbents is influenced by the concentrations of the MB and adsorbent (Fe_3_O_4_@AVS-BC and Fe_3_O_4_@MMT), which can be described by Eq. (5):

5 $${\mathrm{Fe}}_3{\mathrm{O}}_4@\mathrm{AVS}-\mathrm{BC}\&\mathrm{MMT}+\mathrm{MB}\ \left(\overset{k}{\to }\ \right)\ \left\{{\mathrm{Fe}}_3{\mathrm{O}}_4@\mathrm{AVS}-\mathrm{BC}\hbox{--} \mathrm{MB}\right\}\ \mathrm{or}\ \left\{{\mathrm{Fe}}_3{\mathrm{O}}_4@\mathrm{MMT}\hbox{--} \mathrm{MB}\right\}$$

The initial adsorption rate of MB was evaluated using the Elovich model, yielding a value of 2.77 × 10^22^ mg.g^−1^.min^−1^ for Fe_3_O_4_@AVS-BC, which is higher than that of Fe_3_O_4_@MMT (3.44 × 10^4^ mg.g^−1^.min^−1^). The obtained information implies an extremely high initial adsorption rate for Fe_3_O_4_@AVS-BC compared to that of Fe_3_O_4_@MMT and indicates a very rapid adsorption rate for the MB during the initial stages of the process. On the other hand, the Weber-Morris (WM) model exhibited low *R*^2^ values for both Fe_3_O_4_@AVS-BC and Fe_3_O_4_@MMT compared to other models, indicating its inadequacy in describing the adsorption of MB onto these adsorbents. In addition, the multilinear Weber-Morris model (as shown in Fig. 5e, f and Table 4) reveals that the adsorption of MB onto Fe_3_O_4_@AVS-BC occurs over three stages, and the diffusion rate constants *K*_I2_ and *K*_I3_ are lower than *K*_I1_. This suggests that pore diffusion predominantly affects the overall adsorption rate (Zeng &Kan 2021). Conversely, the adsorption of MB onto Fe_3_O_4_@MMT occurs in two stages, and the diffusion rate constant *K*_I2_ is lower than *K*_I1_. Furthermore, the boundary layer thickness (*C*) is 37.43 and 20.31 for Fe_3_O_4_@AVS-BC and Fe_3_O_4_@MMT, respectively, indicating that film diffusion also plays a role in the adsorption process. This confirms the higher adsorption capacity of Fe_3_O_4_@AVS-BC compared to Fe_3_O_4_@MMT.

### Selectivity of the tested adsorbents

**Fig. 6:**
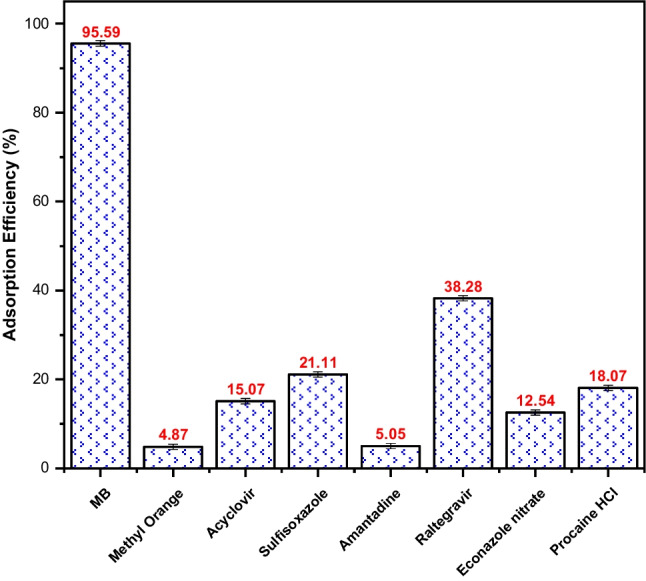
Adsorption selectivity of Fe_3_O_4_@AVS-BC toward MB compared to other organic pollutants

The selectivity of the best-performing adsorbent, Fe_3_O_4_@AVS-BC, was explored by contrasting its removal efficiency toward MB compared to other organic contaminants possessing different chemical structures. Selectivity testing was performed under the optimum experimental conditions for MB as decided upon using the CCD. Figure 6 shows that the performance of Fe_3_O_4_@AVS-BC was the best toward MB with a removal efficiency of 95.59%. This confirms that Fe_3_O_4_@AVS-BC has a high affinity toward the MB molecules, due to specific interactions between the surface functional groups of the adsorbent and MB as proven by the D-R model. Tested interferents showed significantly lower adsorption compared to MB. This could be attributed to various factors, including the suitability of the used factorial blend during adsorption to each pollutant, the pH_PZC_ of the adsorbent compared to the pK_a_ of the adsorbate, and the chemical structure of the pollutant. The highest removal efficiencies were reported for raltegravir and sulfisoxazole, at 38.28% and 21.11%, respectively. It is worth noting that the pK_a_ value for raltegravir for example is 6.30 (Table S2). Given that the pH_PZC_ of the studied adsorbent is 9.85, the surface of the adsorbent becomes positively charged at pH 5. Consequently, raltegravir will also carry a positive charge at this pH, which negatively impacts the adsorption efficiency. On the other hand, removal of the rest of the tested interferents ranged between 4.87% and less than 20%, an issue which reflects the selectivity of Fe_3_O_4_@AVS-BC to MB compared to the rest of the tested interferents. The obtained data suggests that Fe_3_O_4_@AVS-BC is highly selective toward MB and significantly less effective for other contaminants.

### Recyclability of the adsorbent-adsorbate composites

**Fig. 7:**
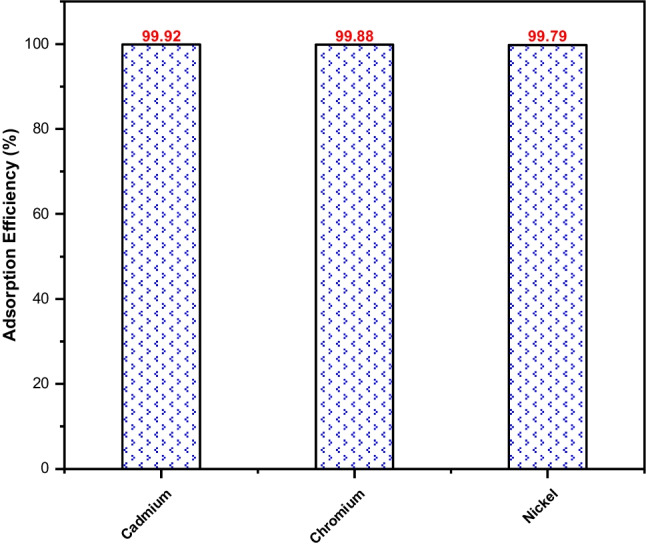
Reusability of Fe_3_O_4_@AVS-BC for the removal of heavy metals from wastewater

The reusability of the MB-laden adsorbent was tested toward another set of aquatic pollutants: Cd (II), Cr (III), and Ni (II). The main objective of this test is to avoid the accumulation of waste (adsorbent-adsorbate composites) following the adsorption process, which is usually a serious concern that affects the applicability of the adsorption on a large scale as a result of secondary pollution. Figure 7 shows an excellent performance of the calcinated composite, MB-laden adsorbent, toward the tested heavy metals with a removal efficiency exceeding 99%. This efficiency could be attributed to the composite multi-site complexation ability, which may result from the presence of specific functional groups on its surface. These functional groups could arise from the existence of the MB on the surface of Fe_3_O_4_@AVS-BC. Moreover, the calcination process could have reactivated the available adsorption sites on the composite surface, allowing for the efficient removal of the heavy metal ions.

## Conclusion

The current study aimed at the removal of MB dye from synthetic wastewater using the biochar of the avocado stones (AVS-BC) as well as the montmorillonite clay (MMT), both in their pristine formats and following their loading with magnetite (Fe_3_O_4_@AVS-BC, Fe_3_O_4_@MMT). The CCD was employed to optimize the variables affecting the adsorption process and maximize the removal efficiency of the tested adsorbents. Due to the superior removal efficiency (%*R*) demonstrated by Fe_3_O_4_@AVS-BC compared to Fe_3_O_4_@MMT (95.59% and 88%, respectively), Fe_3_O_4_@AVS-BC was selected over Fe_3_O_4_@MMT. FT-IR analysis performed before and after adsorption revealed differences in intensities and positions of many functional groups and was used to confirm the successful adsorption of MB onto the surfaces of both adsorbents. Studies of equilibrium have revealed that the results are consistent with Langmuir isotherm. Adsorption of MB onto Fe_3_O_4_@AVS-BC was found to occur via chemical ion-exchange adsorption, compared to physisorption in the case of Fe_3_O_4_@MMT. Kinetic studies showed that the PSO model can be used to describe the adsorption of MB onto both adsorbents. Fe_3_O_4_@AVS-BC exhibited high selectivity toward MB compared to other contaminants. The MB-loaded adsorbent was successfully reactivated via thermal treatment and was successfully utilized for the removal of several heavy metals.

### Supplementary Information

ESM 1: Tables S1-S6 and Figure S1 (DOCX 383 kb)

## Data Availability

All data used to support the findings of this study are included within the article.
