# Supplementary material for: Turning waste avocado stones and montmorillonite into magnetite-supported nanocomposites for the depollution of methylene blue: adsorbent reusability and performance optimization
Source: Environ Sci Pollut Res Int. 2023 Nov 3;30(56):118764–81. doi: 10.1007/s11356-023-30538-0 (PMC10698139; doi:10.1007/s11356-023-30538-0)
Supplement: Supplementary file 1 — Tables S1-S6 and Figure S1 (DOCX 383 kb) [file 11356_2023_30538_MOESM1_ESM.docx]

**Turning Waste Avocado Stones and Montmorillonite into Magnetite-Supported Nanocomposites for the Depollution of Methylene Blue: Adsorbent Reusability and Performance Optimization**

Ahmed S. El-Shafie ^a*^, Fatima Karamshahi ^a^, Marwa El-Azazy ^a*^

^a^ Department of Chemistry and Earth Sciences, College of Arts and Sciences, Qatar University, Doha 2713, Qatar

^*^ Corresponding author at: Department of Chemistry and Earth Sciences, College of Arts and Sciences, Qatar University, Doha 2713, Qatar. Tel.: +974 4403 4665; Fax: +974 4403 4651, e-mail address: marwasaid@qu.edu.qa (Dr. Marwa El-Azazy)

1. **Equilibrium Models:**

Langmuir model suggests the following hypotheses: each site on the adsorbent surface available for the adsorption have single sorption energy, each MB molecule can occupy only single location on the both magnetic sorbent surfaces without any interaction between MB molecules, and MB adsorption occurred mainly on the surface of the two magnetic adsorbents. The favorability of MB adsorption can be assessed by the *R_L_* value, and it can be calculated by dividing the separation factor of MB dye by the MB initial concentration (*C_0_,* mg/L). On the other hand, the Freundlich model provides insights into the adsorbent's capacity, and the disparity in intensity of the sorption process.

The Temkin model is used to explain the type of interaction between the MB and the two magnetic sorbents based on adsorption energy. Finally, Dubinin-Radushkevich (D-R) model was used to estimate MB's adsorption mechanisms (chemisorption or physisorption) onto Fe_3_O_4_@AVS-BC and Fe_3_O_4_@MMT.

**Table S1.** Physicochemical characteristics of MB (Khan et al. 2022, Pandey et al. 2022).

| **Property** | **MB** |
| --- | --- |
| Structure | 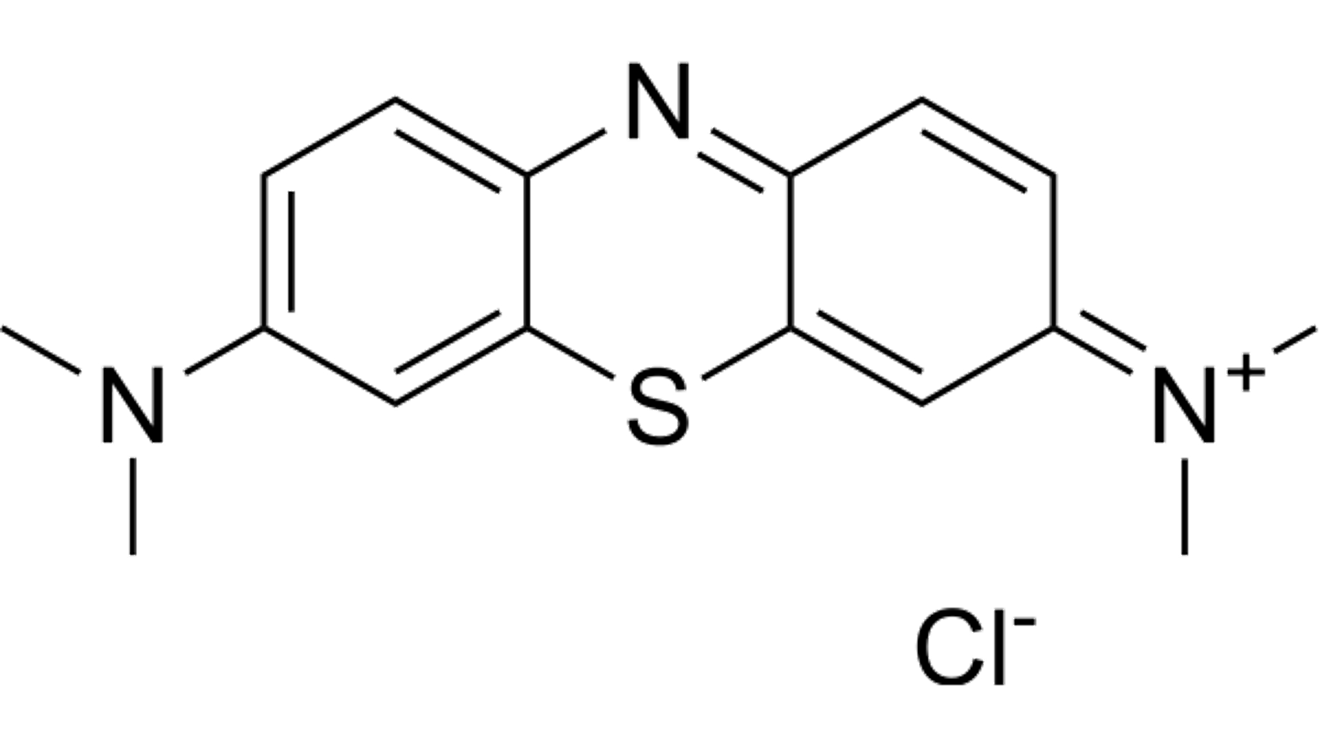 |
| Chemical formula | C_16_H_18_ClN_3_S |
| Name (IUPAC) | 3,7-bis(Dimethylamino)-phenothiazin-5-ium chloride |
| Molecular weight (g/mol) | 319.85 |
| Color index (CI) | 52015 |
| λ_max_ (nm) | 663 |
| pK_a_ | 3.8 |

**Table S2.** Stability and reported pK_a_ values of the studied interferents.

| **Adsorbate** | **pK_a_** | **Chemical Structure** | **Sensitivity to Temperature, pH, or Light** | **References** |
| --- | --- | --- | --- | --- |
| Methyl orange | 3.50 | 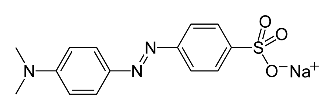 | High for pH and light | (Dirksen et al. 2002, Hanafi &Sapawe 2020) |
| Acyclovir | 2.27 and 9.25 | 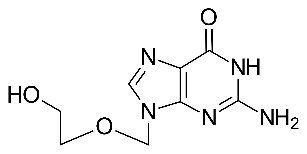 | Stable at room temperature and not stable at higher heat and moisture | (NIH) |
| Amantadine | 10.10 | 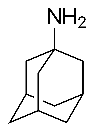 | Stable at room temperature and not stable at higher heat and moisture | (NIH) |
| Raltegravir | 6.30 | 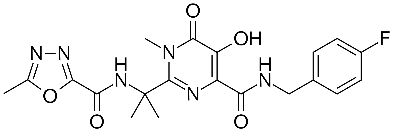 | Stable at room temperature and not stable at higher heat and moisture | (NIH) |
| Econazole nitrate | 6.48 | 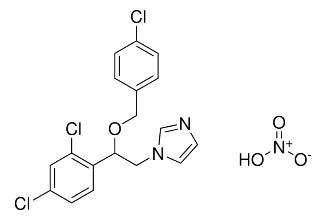 | Stable at room temperature and not stable at higher heat and moisture | (NIH) |
| Procaine hydrochloride | 8.9 | 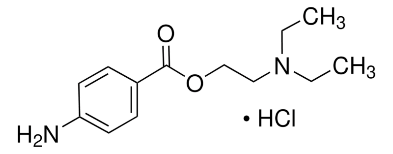 | Unstable in light and air | (Fishersci) |
| Sulfisoxazole | 5 | 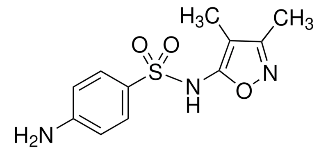 | Sensitive to heat, moisture, and direct light | (mayoclinic) |

**Table S3.** Preliminary experiment for the adsorption of MB onto AVS-BC and MMT before and after loading with magnetic nanoparticles. Experiments were done using the non-optimized conditions: pH = 7, AD = 100 mg, [MB] = 50 mg/L, and CT = 60 min.

| **Adsorbent** | **%R** |
| --- | --- |
| **AVS-BC** | 42.33 |
| **Fe_3_O_4_@AVS-BC** | 72.28 |
| **MMT** | 24.16 |
| **Fe_3_O_4_@MMT** | 52.85 |

**Table S4.** CHN analysis of the tested adsorbents before and after loading of magnetic nanoparticles.

| **Sample name** | **N (wt%)** | **C (wt%)** | **H (wt%)** |
| --- | --- | --- | --- |
| **AVS-BC** | 0.15 | 71.12 | 1.65 |
| **Fe_3_O_4_@ AVS-BC** | 0.12 | 36.17 | 3.63 |
| **MMT** | 0.05 | 0.98 | 1.06 |
| **Fe_3_O_4_@ MMT** | 0.10 | 1.30 | 1.60 |

**Table S5.** Chi-square error function used for the accuracy measurements of equilibrium and kinetics models.

| **Error Function** | **Equation** |
| --- | --- |
| Chi-square (χ^2^) | $\chi^{2}= \sum\left[ \frac{{(q_{exp}-q_{cal})}^{2}}{q_{cal}} \right]$ |
| *Where q_exp_: experimental removal capacity (mg/g), q_cal_: calculated removal capacity (mg/g).* | |

**Table S6.** Nonlinear equations depicting the equilibrium and kinetic models.

| **Equilibrium Model** | **Nonlinear Equation** |
| --- | --- |
| Langmuir | $q_{e}=\frac{q_{m} K_{L} C_{e}}{1+K_{L} C_{e}}$ |
| Freundlich | $q_{e}= K_{F}C_{e}^{\frac{1}{n}}$ |
| Temkin | $q_{e}= \frac{RT}{b_{T}} ln(A_{T} C_{e})$ |
| D-R | $q_{e}= q_{s}$. exp (-β.$\epsilon^{2}$)  $\epsilon=RT(1+ \frac{1}{C_{e}})$  $E= \frac{1}{\sqrt{2\beta}}$ |
| **Kinetics Model** | **Nonlinear Equation** |
| Pseudo first order (PFO) | $\frac{dq_{t}}{dt}$*= k_1_(q_e−_q_t_)* |
| Pseudo second order (PSO) | $\frac{dq_{t}}{dt}$*= k_2_(q_e−_q_t_)^2^* |
| Elovich model | *q_t_=* $\frac{1}{\beta} \times ln(1+\alpha\beta t)$ |
| Weber-Morris (W-M) model | $q_{t}=K_{I}t^{0.5}+C$ |
| *q_m_: maximum adsorption capacity, K_L_: Langmuir constant, K_F_, 1/n: Freundlich constants, A_T_, b_T_: Temkin constants,* *R: universal gas constant, T: temperature, q_s_: saturation capacity, β: activity coefficient, and ϵ: calculated Polanyi potential. q_t_: capacity at t, K_1_: adsorption rate constant, t: adsorption time, K_2_: adsorption rate constant, α, β: Elovich constants, K_I_: intraparticle diffusion rate constant, and C: thickness of the boundary layer.* | |

| 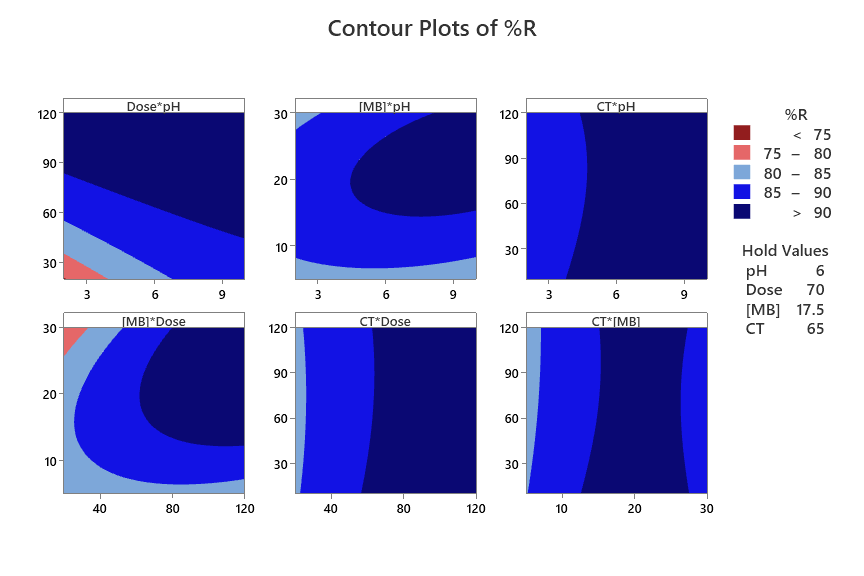  **(a)** | 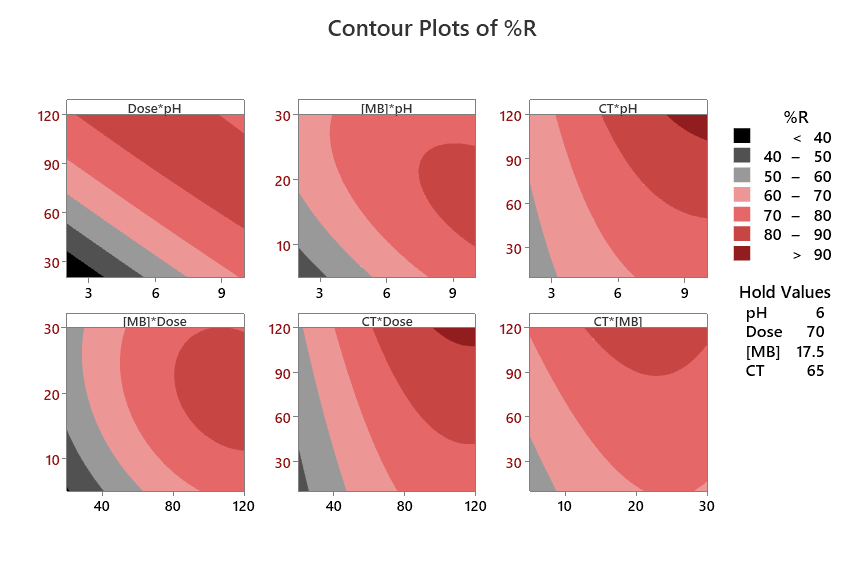  **(b)** |
| --- | --- |
| 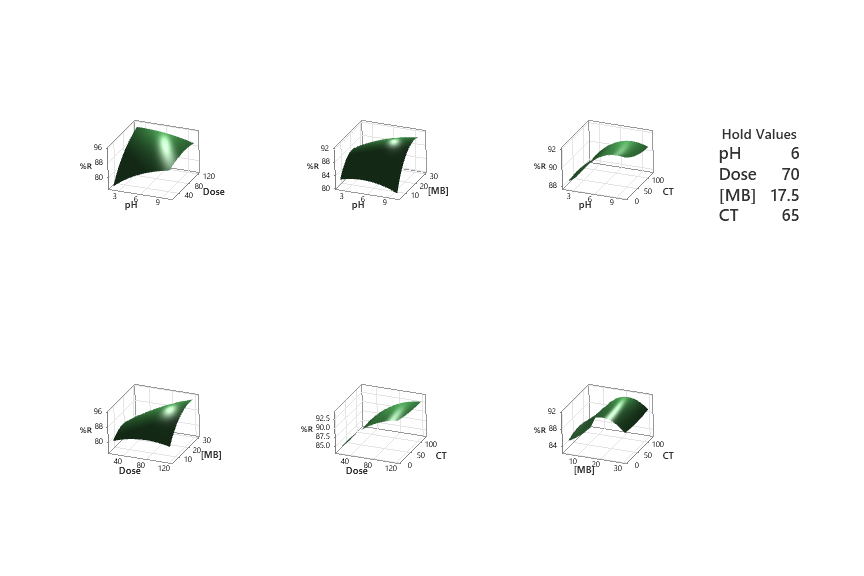  **(c)** | 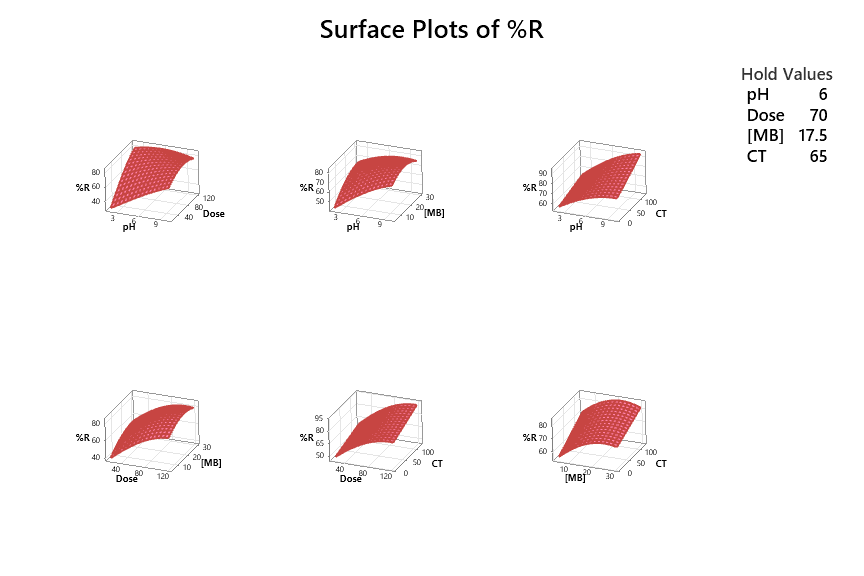  **(d)** |

**Figure S1.** Contour plots **(a, b)**, and surface plots **(c, d)** for the MB %R using Fe_3_O_4_@AVS-BC **(a, c)**, and Fe_3_O_4_@MMT **(b, d)**.

**References:**

Dirksen A, Zuidema E, Williams RM, De Cola L, Kauffmann C, Vögtle F, Roque A, Pina F (2002): Photoactivity and pH Sensitivity of Methyl Orange Functionalized Poly(Propyleneamine) Dendrimers. Macromolecules 35, 2743-2747

Fishersci Material Safety Data Sheet, Procaine hydrochloride. <https://fscimage.fishersci.com/msds/22831.htm>

Hanafi MF, Sapawe N (2020): Influence of pH on the photocatalytic degradation of methyl orange using nickel catalyst. Materials Today: Proceedings 31, 339-341

Khan I, Saeed K, Zekker I, Zhang B, Hendi AH, Ahmad A, Ahmad S, Zada N, Ahmad H, Shah LA, Shah T, Khan I (2022): Review on Methylene Blue: Its Properties, Uses, Toxicity and Photodegradation. Water 14, 242

mayoclinic Sulfisoxazole (Oral Route). <https://www.mayoclinic.org/drugs-supplements/sulfisoxazole-oral-route/precautions/drg-20072998?p=1>

NIH Acyclovir. <https://medlineplus.gov/druginfo/meds/a681045.html#storage-conditions>

NIH Amantadine. <https://medlineplus.gov/druginfo/meds/a682064.html#storage-conditions>

NIH Raltegravir. <https://medlineplus.gov/druginfo/meds/a608004.html#storage-conditions>

NIH Econazole Topical. <https://medlineplus.gov/druginfo/meds/a684049.html#storage-conditions>

Pandey D, Daverey A, Dutta K, Yata VK, Arunachalam K (2022): Valorization of waste pine needle biomass into biosorbents for the removal of methylene blue dye from water: Kinetics, equilibrium and thermodynamics study. Environmental Technology & Innovation 25, 102200
